# Leaving the profession as a medical assistant: a qualitative study exploring the process, reasons and potential preventive measures

**DOI:** 10.1186/s12913-024-11607-7

**Published:** 2024-09-24

**Authors:** Viola Mambrey, Annegret Dreher, Adrian Loerbroks

**Affiliations:** Institute of Occupational, Social and Environmental Medicine, Centre for Health and Society, Faculty of Medicine and University hospital Düsseldorf, Heinrich Heine University Düsseldorf, Universitätsstr. 1, 40225 Düsseldorf, Germany

**Keywords:** Career exit, Germany, Health personnel, Leaving the profession, Medical assistant, Qualitative study, Turnover

## Abstract

**Background:**

Worldwide growing shortages among health care staff are observed. This also holds true for medical assistants in Germany. Medical assistants mainly work in outpatient care and are the first point of contact for patients while performing clinical and administrative tasks. We sought to explore profession turnover among medical assistants, that is, in terms of the underlying decision-making process, the reasons for leaving the medical assistant profession and potential retention measures from the perspective of former medical assistants.

**Methods:**

For this qualitative study, we conducted semi-structured telephone interviews with 20 former medical assistants between August and November 2023. Eligible for participation were medical assistants who (i) were of legal age, (ii) completed medical assistant vocational training and ii) were formerly employed as a medical assistant, but currently employed in another profession. The interviews were recorded, transcribed verbatim and content-analyzed.

**Results:**

Former medical assistants expressed various, often interrelated reasons for leaving the profession. These were changes in priorities throughout their career (e.g., in terms of working hours and salary), a constant high workload, barriers to further training, poor career prospects, and poor interpersonal relationships particularly with supervisors, but also within the team and with patients as well as the perception of insufficient recognition by politics and society. Suggestions of former medical assistants to motivate medical assistants to stay in their profession included amongst others higher salaries, more flexible work structures, improved career prospects, and more recognition from supervisors, patients, and society.

**Conclusion:**

Our study provides insights into the complex decision-making process underlying ultimate medical assistant profession turnover. In light of an already existing shortage of medical assistants, we suggest to further explore how the suggested interventions that aim at retention of working medical assistants can be implemented.

**Supplementary Information:**

The online version contains supplementary material available at 10.1186/s12913-024-11607-7.

## Introduction

Worldwide there is a growing shortage of health care workers [1, 2]. Among the primary drivers are the demographic change in general populations, an ageing health care working population and a general increase in chronic diseases among the general population [3, 4]. The staff shortage is aggravated by the high number of health care workers leaving their profession [5] and the COVID-19 pandemic seems to have exacerbated this trend [6]. Shortages of health care staff are associated with lower patient satisfaction, negative health outcomes for patients, and a decrease in job satisfaction among the remaining health care workers [7]. In addition, the consequences of turnover are the loss of knowledge, productivity and high cost (e.g., temporary replacements, overtime work, less productivity during staff induction) [7, 8].

The majority of studies that have examined potential antecedents of profession turnover and turnover intention among health care staff rely on quantitative designs and thus on numerical data for analyses [7, 9–13]. Compared to qualitative methods, such approaches are unable to capture the complex decision-making process that may underlie profession turnover [7, 14]. Previous qualitative studies among health care workers, particularly nurses, often explored turnover intention or change of employers rather than entirely leaving one’s profession [15]. This is particularly relevant, as one’s intention to leave does not invariably translate into actual profession turnover [9, 10, 16]. So far, studies that profoundly explore the complex decision-making process and circumstances underlying the turnover of health care workers are limited [7, 9, 15]. The qualitative studies that explored reasons for profession turnover among former nurses reported these to include a high workload, poor interpersonal relations with supervisors and colleagues, limited career opportunities, as well as the associated poor mental health [5, 17–20]. These studies, however, focused on former novice nurses or very young nurses [5, 17], were restricted to inpatient care settings [18–20] and were carried out before the COVID-19 pandemic. The COVID-19 pandemic had major impacts on the working life of health care professionals [21, 22].

We seek to address the abovementioned knowledge gaps by exploring profession turnover among medical assistants (MA) who represent the largest occupational group in outpatient care in Germany [23]. MA perform a wide range of tasks in the practice, including administrative tasks and medical procedures such as blood sampling, injections, wound care, X-rays, and laboratory diagnostics [24]. Outpatient care in Germany is generally characterized by work in small teams with the physician often being both the employer and supervisor. MA face a variety of job-specific stressors, such as poor practice organization, interpersonal stressors (e.g., poor collaboration, lack of social resources), or a strong dependence on the supervising physician [12]. There is a concern that the changes in working conditions in the health care system due to the COVID-19 pandemic will lead to increased turnover [6, 25]. This would exacerbate the already emerging trend of MA to migrate to other professions [26]. A prior qualitative study from our group among MA explored the perceived changes of working-conditions associated with the COVID-19 pandemic [25]. MA shared that various stressors have emerged or exacerbated during that period such as an increased workload, changes in collaboration within the team, and an increase in demanding patients. These stressors were reported to elicit intentions to leave the profession [25]. Further, in a recent 4-year prospective cohort study among MA by our group we found in particular interpersonal factors, amongst others poor collaboration within the team, poor behavior of the supervisor to predict turnover from the profession [13]. While that quantitative approach provides data on the predictors of turnover, it fails to provide an in-depth understanding of the underlying decision-making process and the likely interrelationship of reasons for profession turnover among MA. Fostering this understanding is important to develop suitable interventions as part of a comprehensive health workforce policy to strengthen healthcare workers retention and to counteract growing staff shortages, in the case of our study the MA workforce [27]. To gain a profound understanding of how and why MA left their original profession we aimed to explore the decision-making process, the reasons and potential retention measures from the perspective of former MA.

## Methods

### Study design

We carried out a qualitative study building on semi-structured interviews [28]. This particular design was chosen because it allowed a comprehensive exploration of our understudied research topic [15, 28, 29]. The exploration of a broad spectrum of views and the detailed thematic analysis of the data were used to gain an in-depth understanding of the decision-making process, the circumstances and the reasons for turnover from the MA profession.

### Study sample

We conducted semi-structured qualitative telephone interviews using a predefined topic guide. The completed checklist of consolidated criteria for reporting qualitative research (COREQ) were applied in writing this report (see checklist in Appendix 1) [30].

### Participant’s recruitment and interview conduct

We employed convenience sampling. First, we invited former MA from an existing cohort of MA, that is, individuals who had reported employment as a MA at baseline (2016/2017) and reported to be currently employed, but not as a MA at follow up (2021) (*n* = 48) [31]. Second, MA were recruited through the professional organization of medical assistants in Germany (“Verband medizinischer Fachberufe e.V.”) which advertised our study on their social media profiles (Instagram, Facebook) on November 9th, 2023. Third, MA were recruited through personal contacts. Inclusion criteria were the legal minimum age (i.e., 18 years in Germany), completion of the MA vocational training, and that participants had formerly worked as a MA and were currently employed in another profession. The latter was important as we specifically wanted to explore the views of MA who had chosen to leave their profession (in contrast to MA in retirement or on parental leave) and would theoretically still be available for the MA workforce. Potential participants received written study information and provided written informed consent prior to participation. Recruitment and interviewing lasted from August 14 until November 28, 2023. The sample size was not determined a-prior, but was contingent upon thematic saturation. It has been suggested that saturation is, most often reached between 9 and 17 interviews in qualitative interview studies [32]. An external contractor transcribed the recorded interviews verbatim while omitting potentially de-anonymizing information from the transcripts. Interviews were conducted in German and quotes were translated into English by a research associate with a Master’s degree in English Studies. The ethics committee of the Medical Faculty of the University of Düsseldorf approved the study (study number 2023–2443).

### Development of the topic guide

The topic guide was developed based on an extensive review of pertinent literature on turnover particularly in health care, which emphasized the significance of understanding the career decision-making process [7, 10, 11]. In order to achieve this understanding, relevant dimensions of the process need to be explored: the initial decision for the profession, the circumstances around the turnover, potential triggers or challenges regarding the leaving process, and general aspiration for one’s future career [7, 10, 11]. The topic guide was designed to explore the process of how MA exit from their profession, including the circumstances of quitting, criteria for the selection of the new workplace and potential starting points for measures to prevent MA turnover. The initial topic guide was discussed within the study team and revised until consensus was reached. VM, AD and AL are occupational health researchers and have experience in qualitative research, the development of topic guides and qualitative data analysis, including qualitative studies among the MA population (for the topic guide see Appendix 2) [25, 33]. Moreover, in the qualitative interviews, we used 13 closed-ended questions to collect data on socio-demographics (i.e., sex, age, partnership, highest educational degree, recruitment channel), work-related information (i.e., years worked as a MA, time passed since having left the MA profession, former employer [i.e., general practitioner (GP), specialist, others], current employment in the health sector, and perspectives on the MA profession. The latter was assessed as follows: (a) likelihood of recommending younger people to take up the MA profession; (b) to what extent they would decide to take up the MA profession as of today; (c) probability of returning to the MA profession; and (d) satisfaction with their current job as compared to their job as a MA). Appendix 2 shows the final topic guide including the close ended questions.

### Data analysis

We conducted our analysis building on Kuckartz’ content structuring content analysis using MAXQDA 2024 software (VERBI GmbH, Berlin, Germany) [34]. Categories were formed deductively according to the research questions. The main categories were then refined by inductively forming subcategories from the interview transcripts. The first five interviews were coded independently by VM and AD. The preliminary coding scheme was then compared and discrepancies were discussed until consensus was reached. The resulting coding scheme was then applied to code the remaining transcripts (VM). After the first full round of coding the coding scheme was reviewed by AL. A second round of coding was performed by VM with consideration of the suggestions.

## Results

### Sample characteristics and descriptive results

In total, we conducted 20 interviews. Participant characteristics are shown in Table 1. Most participants were recruited from our MA cohort study (*n* = 14). The interviews had a mean duration of 38.1 min ranging from 23 to 66 min. We interviewed 18 female participants and two male participants with a median age of 44.5 years ranging from 26 to 64 years. The participants had worked as a MA for a median of 22 years (min-max: 5–41 years) and the median number of months since the last exit from the MA profession was 40.5 (min-max: 10–132 months). The majority would somewhat to fully recommend the professional training as a MA to (young) people (*n* = 14), but more than half of the interviewed former MA would rather not or certainly not choose the MA profession again if they had to make that decision again (*n* = 11) (see Table 2). All of the participants reported that a return to the MA profession was unlikely or very unlikely.

**Table 1:** Descriptives of the sample (*n* = 20)

Characteristic	
Sex, n	
Female	18
Male	2
Age, median (min-max)	44.5 (26–64)
In a partnership – yes, n	17
Highest school degree^a^, n	
Low	0
Intermediate	10
High	10
Years worked as MA^b^, median (min-max)	22 (5–41)
Month since last exit from MA profession, median (min-max)	40.5 (10–132)
Last employment as a MA, n	
General practitioner	6
Specialist	10
Others (rehabilitation center, emergency room, practice in private clinic)	4
Working in health sector, but not as MA, n(%)	13
Way of recruitment	
MA cohort study^c^	14
Professional organization of MA^d^	5
Personal contact	1

^a^ low: secondary school qualification (‘Haupt-/Volksschulabschluss’); intermediate: secondary school level I certificate (‘Mittlere Reife’); high: general qualification for university entrance (‘Abitur’) or entrance qualification limited to universities of applied sciences (‘Fachhochschulreife’)

^b^ Medical assistants (MA)

^c^ participants reporting employment as a MA at baseline (2016/2017) and currently employed, but not as a MA at follow up (2021) were eligible (*n* = 48)

^d^ advertisement on their social media profiles (Instagram, Facebook)

**Table 2:** Agreement to job-related questions about the former profession as a medical assistant (MA)

Characteristic	*n*
The following question relates to the professional training as a MA: I would recommend the professional training as a MA to (young) people.	
Do not agree at all	3
Somewhat disagree	3
Somewhat agree	10
Fully agree	4
If you had to make the decision again, would you choose the profession of MA again as of today?	
Certainly not	6
Rather not	5
Probably yes	7
Certainly	1
Don’t know	1
How likely do you think it is that you will return to the MA profession?	
Very unlikely	9
Unlikely	11
Likely	0
Very Likely	0
Are you more satisfied with your current job than with your job as a MA?	
Yes	17
No	1
Neither nor	2

### The decision-making process: changing priorities and barriers to quitting

#### Entrance into the MA profession

Participants described that their decision to take up professional MA training was often based on first-hand experiences with the profession through internships as a student or after graduating from high school, based on pragmatic reasons (e.g., a workplace close to one’s home, doing any kind of vocational training) or perceived as a coincidence (i.e., “it turned out that way”). Expectations prior to the training were to work in a medical profession, to work closely with people and to carry out administrative work. Some former MA remembered not having any expectations, which was often attributed to their young age and thus their inexperience at the start of training.

#### Barriers to quitting

Many former MA expressed that they enjoyed working as a MA, because of their personal interest in medical topics, the variety of tasks, and the close work with patients. Participants described, however, a discrepancy between these expectations and experiences towards and during their MA job at a young age and their personal needs and priorities in later working life (e.g., higher salary, work-life balance/family friendly working hours, better career prospects). Many emphasized that their decision to quit the MA profession was carefully considered and not made hastily. In fact, quitting was perceived to be often delayed due to personal reasons (e.g., low self-confidence with regard to one’s skill and career prospects, not seeing any alternative career path for oneself), financial reasons (e.g., being dependent on the salary as a single parent), a sense of loyalty towards colleagues and the employer/supervising physician. In addition, participants felt that their exit from the profession was delayed, because the physician attempted to keep MA down (compare verbatim quote Q1 below). Further, they felt ”emotionally blackmailed” by their supervising physician who took advantage of the loyalty of MA towards their colleagues (i.e., higher workload for colleagues if MA leaves due to staff shortage).

“I had to earn money. So, there was a total interdependency, and one was afraid, or one wasn’t confident enough to say: ‘I’ll find something new in another medical practice.’ It is especially your bosses who convey this feeling of dependency to you: ‘You’ll never find a new job if you quit now.’ This is really exhausting. And as a young woman, you actually believe that and then you’re too afraid to quit.” (Q1, ID 7123; for verbatim quotes see Appendix 3).

Some participants felt that changing the profession was like a natural progression in their career. By contrast, for others it felt like a difficult decision. The experienced working conditions as a MA made them feel that merely changing their employer (while continue working as a MA) would not have improved their situation. The mainly positive experiences they had made in the new profession and the disbelief that MA working conditions will improve soon, led most participants to believe that they would not return to the MA profession.

### Reasons to exit from the MA profession

The former MA reported various reasons to quit their profession and emphasized that there were often multiple reasons (for an overview of the reasons for MA profession turnover see Appendix 4).

#### Constantly high workload

Many described that a constantly high workload was a key reason for their decision to quit. Different facets of such a high workload were mentioned: First, the reasons for a high workload were mentioned. These were, first, a high number of patients and the limited time to fulfill tasks and adequately care for patients. This made participants feel stressed and emotionally blunted (Q2, for verbatim quotes see Appendix 3). Moreover, the high number of patients made it necessary to triage patients on the phone according to the perceived severity of symptoms. Frequently, having to turn patients down on the phone or discussing with them how urgent their complaints were (not) was perceived as stressful (Q3). A second reason for the perceived increasing workload that contributed to quitting were increasing administrative tasks (e.g., accounting, documentation, data protection). Participants often felt that documentation was at the core of their work rather than working with patients.

“And eventually, it was not about the patients anymore, you just had to fill in more and more documents.” (Q4, ID 6912).

A third perceived contributor to the high workload were cumbersome and outdated processes (e.g., slow digitalization, faxing, necessity to print documents), which were experienced as time consuming, highly inefficient and frustrating, because time was perceived as scarce (Q5).

Aside the causes, participants also explained the consequences of the high workload. A high workload was perceived to result in long waiting times for the patients which was felt to translate into substantial dissatisfaction among patients and into working overtime for the MA. The perceived consequences of a persistent lack of time due to a high workload were (i) an inability to provide adequate patient care; (ii) lacking opportunities to expand medical knowledge during working hours (e.g., discussing patients with physicians); (iii) often not having a single break throughout the work day and (iv) feeling stressed. Work stress was felt to lead to physical and psychological complaints (e.g., back pain, burnout) and negatively influence the team atmosphere (Q6).

“It’s getting more and more. It’s not decreasing and it’s not stagnating either. On the contrary, requirements have increased more and more, but the salary certainly hasn’t. The way we treat each other in the team has also become less pleasant, because we all reach our limit at some point.” (Q6, ID 6734).

Long working days due to the high workload and the operating hours of physician offices with long midday breaks, often including (expected daily) overtime, in some cases even unpaid, were felt to be frustrating (Q7). These consequences of a high workload were mentioned as factors contributing to the decision to leave the MA profession.

Finally, concerning a high workload, former MA felt that there was a shortage of skilled MA which leads to a vicious cycle of - on the one hand – increased stress-induced absenteeism, which then reinforces staff shortage and increases the workload for the remaining MA (Q8, Q9). On the other hand, the lack of skilled MA was perceived to result in constant spontaneous changes in working shifts without the workload being adapted to the reduced workforce (e.g., rescheduling appointments), negatively affecting the private life (Q10, Q11).

#### Perceived barriers to further training and poor career prospects

Many former MA expressed that the limited further training and career prospects made them leave the MA profession (Q12). Participants felt that the missing career prospects had led to a sense of missing challenges and increasing boredom.

“I don’t want to spend another 20 years printing out prescriptions, talking to people on the phone and making appointments.” (Q13, ID 9162).

One former MA criticized that although MA could acquire skills through further training, they often did not have the legal permission to apply these skills. This means for example that they are not allowed to act as a formal training supervisor for MA in training. This is the sole legal responsibility of the physician in Germany. This was perceived as generally unfair and criticized as physicians do not have to complete any relevant training. Others felt the need to leave the MA profession as they perceived that the further training and novel skills could not be applied (Q14). On the one hand, they mentioned limited demand and thus restricted opportunities to apply their skills particularly in rural areas and/or small practices. On the other hand, the limited willingness of the employers to pay according to the acquired qualifications was mentioned, which was partly seen to be due to restricted financial leeway (i.e., small practices with limited revenue).

“The stagnation. In other words, I didn’t have the chance to have more responsibility, or earn more money anywhere.” (Q15, ID 9162).

Moreover, the costs for further training were often not or only partially covered by employers. Alternatively, coverage of fees was subject to conditions (e.g., continue working in the practice for five more years). Such conditions were not well received according to participants as further trainings were often only offered during their leisure time (i.e., Wednesday afternoon, weekends), were expensive in relation to the MA salary and were perceived to ultimately benefit the practice.

#### Interpersonal factors

##### Supervisor

A core theme that emerged from the interviews was the perceived behavior of supervisors towards MA as a reason for leaving the MA profession. This included insufficient recognition, poor support, exploiting dependency relationships and disrespectful/aggressive behavior. Many participants expressed to not have felt adequately appreciated by their supervisor(s). In this respect the salary was often alluded to which was perceived as insufficient and unfair in light of the responsibilities of MA, also in comparison to similar healthcare professions (e.g., nurses, nurse assistants) (Q16). They felt they were unable to financially support themselves (and their family) despite working full-time. Some MA only became fully aware of the low salary later in their working life.

“Back then, I couldn’t imagine that the money I was getting was very little. So, I didn’t realize that you can’t actually live off the salary of a medical assistant.” (Q17, ID 8982).

Participants frequently criticized that employers were not obliged to apply the collective wage agreement^1^. Many reported that the contracts were only “based on” the collective agreement, therefore not legally bound by it and often paid less. Further, even if strictly applied, wages according to the collective wage agreement were nonetheless perceived as insufficient as everyday “amenities” like owning a car or buying a pair of new shoes could not be afforded (Q18, Q19). The collective agreement stipulates that employees are assigned to different salary categories based on their qualifications and years of employment. Former MA said that they were not grouped correctly within the collective agreement according to their qualifications and years of experiences and respectively were paid less than they were entitled to which contributed to exit from the profession (Q20). Some former MA acknowledged that considering the profits that employers made under the current remuneration system applying to outpatient physicians, a higher salary for MA would be difficult to implement. Moreover, participants expressed that as long as other work-related factors were satisfactory, these seemed to compensate a salary that was perceived to be inadequate.

Former MA also shared that they felt that physician supervisors considered themselves as superior, that they felt that MAs’ performance and professional skills remained unrecognized and they were disappointed about the lack of their say within the practice.

“For me, it was mostly about the lack of recognition of what you were doing there whatsoever. […] It’s hectic, it’s chaotic and so on, but that has never really been an issue. For me, it has always been the lack of recognition, at least at the end.” (Q21, ID 8463).

The latter included, amongst others, that employers/supervising physicians decided without conferring with MA when to take their vacations. This implies that the practice remains closed during that period of time and MA, consequently, had to take their own vacation, too (i.e., leaving no remaining vacation days for individual vacation planning). Further, there was a perceived lack of say with regard to decisions on practice organization (e.g., communicating with MA how processes in their work areas can be optimized, instead of supervisors deciding alone) (Q22). In addition, a lack of support from supervisors for MA made them feel left alone and overwhelmed with the responsibility in certain situations (e.g., writing medication plans without supervision, training newly hired MA, implementing new measures), and lack of support for MA in front of patients was even perceived as degrading by one MA (Q23).

Some participants mentioned the strong dependency relationship between supervisors and MA as problematic. It resulted in the feeling that MA are kept down and feel to be at their supervisor`s mercy (e.g., things are promised during the job interview, but are revised after the start of work; not being given any responsibility; the range of tasks not corresponding to the MA training).

“[…] Well, in the practice […] our cleaner quit. And then my colleagues started cleaning the practice. And I was like: ‘Are you crazy?’ I would never do that. Not because I consider myself too good for cleaning. I’ve earned a lot of additional money with cleaning. But they hired me as a medical assistant and not as a cleaner, so there’s a limit there.” (Q24, ID3647).

They felt restricted in their capabilities to take action against these conditions, as supervisors are often also the employer and legal protection against dismissal is low in small companies with less than 10 employees. This motivated some former MA to change into the public service sector or to larger companies where employees’ rights are better protected.

Former MA alluded to overemphasized economic thinking of the employer as a further reason to quit. This included that employers wanted to limit the time of social interaction between MA and patients (e.g., on the telephone) due to economic reasons (Q25). Also, MA in training may be hired instead of trained MA to reduce staff costs. Participants felt that the focus was on economic efficiency rather than quality of patient care or wellbeing of the employees (Q26). They found this disappointing and perceived this as dissonant with their reasons as to why they chose the MA profession in the first place (Q27).

##### Colleagues

For some former MA, strong differences of opinions within the team and a constantly tensed team dynamic like bickering, lack of identification with MA colleagues, bullying, non-constructive team meetings were reasons for changing the profession (Q28).

##### Patients

Former MA frequently expressed that the patients’ pronounced (and increasing) demanding behavior (e.g., immediate receipt of prescriptions or appointments, unwillingness to wait, impudence, lack of consideration for other patients and work processes) sometimes combined with aggression, was perceived as stressful, frustrating and to strongly reduce work satisfaction (Q29).

“Expectations have become much higher in the last years. That means, they want something, and they want it right now. And I’m not talking about people who lost their arm, you know? […] Rather, these are people who need a prescription, […] They forget about it, but we have to do it right away. And then, you always have to ask yourself: ‘Alright, should I argue with that person or should I just do it?’ And then you try to make your position clear and tell them: ‘Please let us know at least one day in advance.’ Well, somehow you always have to discuss it. ‘Well, can’t you just quickly do it on the side?’” (Q30, ID5975).

Further, former MA discerned a lack of recognition from patients towards MA and the MA profession, amongst others not being perceived as medical personnel, questioning of MA professional competence (Q31).

#### External factors

Former MA also referred to external factors which influenced their decision to leave the profession. For instance, politics were mentioned in terms of the inadequate legal frameworks they provide which was perceived to ultimately influence MA work and/or working conditions (e.g., only simple tasks can be delegated to MA by the supervising physician, budgeting of health services by the statutory health insurances, which forces employers to think more economically and adversely influences salaries paid by them to MA). Moreover, former MA perceived a lack of recognition from society for the MA profession in general and in comparison to other health care professions such as nurses and physicians. For some former MA the feeling of low recognition by society and politics became very apparent during the COVID-19 pandemic. For instance, despite their exceptional commitment (e.g., being “COVID-19 experts” for patients, being constantly exposed to SARS-CoV-2 positive patients, working overtime), they did not receive COVID-19 financial bonuses like many other professions in health care in Germany or workers in other areas who worked from home (Q32, Q33).

“Well, people who were at home got it. (laughs) [note: single payments during the COVID-19 pandemic] And I’ve been there for ten hours instead of eight, and people giving me a hard time for ten hours and I simply didn’t get it.” (Q33, ID 5975).

### Potential interventions

Former MA suggested several interventions that may help to motivate MA to stay in their profession. Table 3 provides an overview of the addressed actors (e.g., employers, fellow MA, policy makers), the interventions and examples of how former MA thought the interventions can take shape. Participants mentioned many potential interventions that directly address the specific reasons for quitting. These include, amongst others to reduce staff shortage, to increase recognition of MA, to increase the salary, to strengthen supervisor’s leadership skills, to create better career prospects, and to address demanding behavior of patients. Three interventions addressed aspects not specifically mentioned as a reason to quit, but were believed to be particularly feasible and effective for staff retention. The first addressed supervisors and suggested to provide more work flexibility to MA by offering working from home for administrative tasks. The second addressed politics by proposing to increase the quality of the MA training (e.g., oblige supervising physicians to teach certain learning content, differentiate according to medical specialty), which was perceived to indirectly strengthen the MA profession. The third intervention suggestion emphasized that the MA themselves should strengthen the MA profession by standing up for themselves more, engaging more in networking and encouraging each other to talk about the terms of their contracts.

**Table 3:** Potential preventive measures to motivate MA to stay in the MA profession

**Actors**	**Intervention area**	**Potential interventions**
**Employers/ supervisors**	Address staff shortage	• In case of staff shortage: cancel patient appointments
	Implement more flexible working hours	• Consider working time preferences of MA • Shorten working days by shortening long lunch breaks • Offer various working time models (e.g., part-time, four-day week) • Offer family-friendly working hours (e.g., not working late afternoon/evenings)
	Provide more work flexibility	• Allow MA to work from home for administrative tasks • Offer more quiet working options (e.g., working from home, back office)
	Increase recognition of MA	• Express recognition for MA (e.g., show interest in the MA, acknowledge MAs’ work and medical expertise) • Include MA in decision-making processes (e.g., consider suggestions for change/improvements, planning vacation times together)
	Increase salary	• Pay according to collective agreement or at least above minimum wage • Pay according to qualification and years of experience • Provide bonuses based on the financial success of the practice • Increase salary or make one-time payments instead of giving gifts • Adjust salary automatically (i.e., if not part of collective agreement) • Bear cost for further trainings
	Strengthen leadership skills	• Participate in courses on leadership • Behave supportively towards MA in front of patients • Adjust distribution of tasks if necessary (i.e., avoidance of work overload for individual MA)
	Strengthen team cohesion	• Commit to team-related matters (e.g., resolving team conflicts) • Organize team activities (i.e., familiar atmosphere) • More team - less hierarchy (e.g., develop solutions together as a team) • Create work councils (i.e., solve problems more objectively) • Do not employ spouse in practice (i.e., often not trained as MA, power imbalance within team) • Employ/train more male MA (i.e., improved team harmony, less bickering expected)
	Support work independency of MA	• Transfer responsibility to MA (e.g., MA can implement acquired skills on their own)
**Employer association of MA employers **	Increase salary of collective agreement	• Adjust salary to public service collective agreement • Set stricter frameworks for salary adjustments after further training
**Politics**	Create (more) career prospects	• Expand range of tasks and consequently areas of responsibility (e.g., broaden MAs legal capacity, allowing to provide medical advice; more demanding [i.e., more in-depth] further training measures) • Improve accessibility to further training (e.g., offer higher number of further trainings; communicate further trainings better, offer further trainings during working hours; reduce costs of further trainings or offer for free) • Strengthen physician assistant bachelor study and expansion to other specialist areas
	Lower economic pressure of employers	• Reform billing of services (e.g., adjust fixed rates, facilitate billing of provided services, facilitate billing of services from MA, adjust for inflation) • Subsidize MA salary (e.g., care assistants in inpatient care) • Equate/subsidize tax reliefs for MA (e.g., inflation adjustment, COVID-19 bonus)
	Reduce number of administrative tasks	• Reduce regulations • Reduce separate applications • Increase efforts to improve implementation of new processes
	Driving forward modernization of practice processes	• Increase (financial) pressure on the outpatient care employers
**Unspecific actors**	Increase recognition in society	• Strengthen attractiveness of MA profession (e.g., improve career prospects, communication via media) • Communicate MAs’ range of task (e.g., job title is MA and not receptionist)
	Increase quality of MA training	• Increase quality of learning content • Oblige supervising physicians to teach certain learning content • Differentiate according to medical specialty • Separate MA training into administrative or patient-oriented MA
	Address demanding behavior among patients	• Raise awareness of demanding behavior of patients (e.g., explain/communicate financing and budgeting in the healthcare sector, communicate MA shortage and consequences for practice processes including wait times)
**MA themselves **	Strengthen MA profession	• Engage more in networking • Stand up more strongly for oneself (e.g., concerning demands to supervisors) • Reaffirm to talk about contracts

## Discussion

To the best of our knowledge, this is the first study exploring the decision-making process and reasons for leaving the MA profession as well as potential intervention measures. Our findings suggest that MA leave their profession often due to a combination of several factors. We found that expectation towards one’s profession and working conditions at the start of the career change throughout one’s working life and expectations or needs in later working life are felt to remain unmet. The main reasons for leaving the MA profession mentioned were a persistently high workload, barriers to further training and career advancement, poor interpersonal relationships especially with supervisors, but also with patients and within the team as well as poor recognition by politics and society. In order to motivate MA to stay in their profession, participants suggested higher salaries, addressing staff shortage, lower economic pressure and increasing recognition from supervisors, patients, and society.

### Comparison to prior research

#### The decision-making process

Former MA perceived a discrepancy between the expectations they had when they chose their profession (e.g., to closely work with people and help them) and reality (i.e., no time to adequately care for patients) as well as the change of priorities throughout the career, (e.g., related to the salary, working times and career prospects). These findings are in line with those from a qualitative study that explored the decision process underlying turnover among nurses in Australia: that study found nurses to leave due to what the author referred to as “value images violations” indicating a mismatch of expectations vs. reality and “violation of trajectory and strategic images” indicating a change in priorities [35]. According to the author, nurses who perceived a mismatch of expectations vs. reality were usually older with more years of work experience, were in nursing as their career of deliberate choice and struggled tremendously with their decision to leave. By contrast, the decision process for those reporting changes in priorities was characterized as easy and fast [35]. As part of the decision process former nurses who obtained a university/college degree often reported a discrepancy between the nursing taught in class and how it is in practice after entering the profession [5, 17, 20]. This experienced discrepancy right after entering the profession was not mentioned by the participating former MA. This might be explained by the fact that MA training in Germany is based on a dual training program, with trainees mandatorily alternating between vocational school and work in a practice and thus already gaining professional experience as a MA [36].

#### Reasons for leaving the MA profession

Further, in line with our findings, other studies found that often a combination of factors lead to turnover and that single events trigger the decision to leave only infrequently [5, 17]. Overall, it seems that many former MA perceived the low salary as a drawback of the profession. As long as other work-related factors were satisfactory, these seemed to compensate the salary. Once former MA perceived other factors were not rewarding anymore (e.g., career prospects, recognition, sufficient time for good quality patient care) these were often the tipping point leading to turnover. This highlights the complexity of the turnover decision process.

One of the core themes that emerged were high (and increasing) patient demands including rude and impatient behavior as reasons for turnover. In qualitative studies among former nurses, the demanding patient care itself [5], demanding behavior of patient relatives [18] and violence of patients [17] were reported as reasons for leaving the nursing profession. Only one study reported increased expectations of patients and their relatives as a factor perceived to affect turnover [37]. The authors of the latter study used open-ended questions to assess perceived antecedents of turnover among working hospital nurses in Australia. In a qualitative study from our group among MA in 2015/2016 on work-related stressors and resources we found that some experienced MA had perceived a change in patients’ attitudes and expectations towards more impatience [33]. During the COVID-19 pandemic, we carried out another qualitative study and observed that MA perceived a change (compared to the pre-COVID-19 era) in patients’ behavior towards demanding, selfish and aggressive behavior [25]. This might be due to patients becoming more consumer-oriented, with greater expectations of active involvement in their health care as well as greater emphasis on convenience in their health care experiences [38]. This might act as a barrier in the care of patients, if patients perceive healthcare as a service like any other (e.g., restaurant) [39]. MA most likely will not be able to comply with these heightened expectations given the current structures in the German health care system.

Qualitative studies on turnover among nurses from inpatient settings have suggested that a lack of support from the supervisor [19] as well as an occupational culture of hierarchy and discrimination are reasons for leaving the profession [15]. In a qualitative study among nurses from Canada who have left their position, the nurses expressed to be hesitant to voice concerns towards supervisors in fear of being penalized [19]. Further, a perceived lack of respect of physicians towards nurses was reported [19]. This is similar to the accounts of former MA in our study that emphasized strong hierarchical structures and feelings of subordinance towards supervising physicians to contribute to turnover. However, in addition to the lack of support from supervisors as well as the hierarchy, former MA also felt a strong relationship of dependency on their supervisors. The fact that the supervisor is often also the employer creates for some MA the perceived challenge that they constantly have to prove themselves as well as a feeling of restricted means to counter this condition due to a fear of punishment. The dependency relationship is enhanced as, employers in small companies (< 10 employees) – such as outpatient practices – face reduced legal hurdles in terminating employment in Germany. This also means that MA can be dismissed without any severance pay. Further, without a work council, there are often no formal contact persons who can mediate in the event of arguments. As a result, many MA working in outpatient care switch to inpatient care (e.g., offering higher salaries, more flexible working hours, and a work council) or leave the profession [40].

Despite the differences between MA and nurses in terms of their job profile and settings (i.e. outpatient vs. inpatient), former MA reported similar antecedents of turnover as nurses from different countries (e.g., high workload, limited career opportunities, interpersonal difficulties and a low salary) [5, 17–20, 37]. However, we found some factors, which seem to be more specific to outpatient care (e.g., dependency relationship between MA and supervisor), and factors that potentially became more prominent in recent years (e.g., demanding behavior from patients).

### General situation of MA in Germany

Former MA criticized the economic thinking of employers, as in their opinion it contradicts their expectation of providing good patient care. Former MA in our study seemed to become aware that the practices are commercial enterprises though. This might be due to the close cooperation with the employers, the small company structures and because MA are also responsible for the accounting [24]. MA felt that their salary is too low. Notably, general physicians, too, seem to perceive the opportunities to paying higher salaries to MA to be limited by the current remuneration system applying to outpatient physicians [41]. Former MA also believed though that the remuneration of the practices was high and, together with the lifestyle of the employers (e.g., expensive vacations, cars), this created feelings of injustice. The disappointment seemed to prevail specifically during the COVID-19 pandemic as MA perceived their contributions were high and did not translate into bonuses or adaptations of salaries [25, 42]. Likewise, the focus on being an economic enterprise seemed to elicit so-called “moral distress” among former MA, as they felt they have too little time to adequately care for the patients. Moral distress describes a state in which one knows what would be the ethically right thing to do, but feels prevented from acting accordingly [43]. Moral distress has been hypothesized to be experienced to a higher degree among more experienced nurses [44] and so far, has not been specifically studied among MA.

### Qualitative vs. quantitative approaches to turnover

In a four-year prospective quantitative study from our group, we found psychosocial working conditions particularly reflecting interpersonal relations (i.e. poor collaboration, lack of social resources and poor leadership behavior) rather than work demands and resources (e.g., high workload, low job control, poor practice organization) to predict turnover among MA [13]. Those findings are consistent with the results of this qualitative study. However, at the same time, it becomes evident that the quantitative assessment is limited in its ability to uncover the complex underlying decision-making process and the multi-layered reasons and their interaction of MA turnover that emerged from this qualitative study (e.g., not a generally high workload itself, but a perceived constantly increased workload being important for the decision of turnover; low salary being compensated by otherwise good working conditions, however, a change in those leading to turnover). Moreover, in the qualitative interview’s themes emerged, like e.g., high patient demands, moral distress, which were not captured by the quantitative assessment and could thus not be examined as potential determinants of MA turnover.

### Potential intervention measures

In this study, former MA suggested several potential interventions addressing different actors. The proposed interventions addressing politics and unspecific actors are to be implemented on a macro level (i.e., societal) and potentially require longer implementation periods. These are in line with the “global strategy for human resources for health: Workforce 2030” by the World Health Organization [27]. That strategy proposes the implementation of policies on country level to shape health labor markets by addressing education and further training of health workers, the retention of health workers, the inefficiencies of productivity such as pronounced administrative tasks, and the promotion of better working environments [27]. The measures proposed to the supervisors are located on a micro level (i.e., individuals, one-on-one interactions) or meso level (i.e., organizational level) and might prove to be easier and faster to implement than macro level interventions. A qualitative study from our group explored work-related intervention needs of MA and how these could be addressed according to GPs. In terms of an increase of expressed recognition and strengthening of leadership skills, GP perceived deficits however, only among other GPs and not themselves [41]. Many GPs did not perceive that they could improve their expressed recognition towards their MA. At the same time though, the GPs acknowledged that it is their responsibility to improve leadership skills and proposed participation in leadership courses. In contrast, in our study, former MA suggested several intervention areas that the supervisor could address easily, amongst others to strengthen team cohesion; to support work independency of MA, to allow working from home (compare Table 3). Further, former MA proposed that supervisors should actively express recognition for MA by showing interest in the MA and acknowledging MA work and medical expertise as well as include MA in decision-making processes (e.g., accept suggestions for change/improvements, planning vacation times together). It thus seems that the expression of recognition could be achieved more easily by supervisors than they assume in order to reduce the likelihood that MA leave the practice and the profession as a whole (e.g., acknowledge MA work compared to taking a leadership skill course). This includes an open dialog with MA on what MA perceive as recognition. However, it needs to be mentioned that employers are limited in their scope of action to address the understaffing and corresponding high workload of MA due to the already existing shortage of MA [45].

### Methodological considerations

A strength of this study is that we included participants who actually left the MA profession rather than changed employer or merely had the intention leave. We interviewed both female (*n* = 18) and male former MA (*n* = 2) (98% of the general MA profession is female) [46]. Further, we included former MA from different (practice) settings (e.g., GP, specialist, rehabilitation center) as well as a broad range of years of work experience (ranging from 5 to 41 years), thereby likely exploring a large scope of potential views.

Nevertheless, this study has some limitations. First, we cannot rule out a recall bias. Most of the former MA left the profession three years ago at the time of the interviews (median = 40.5 months). This time lag could have influenced the participants’ perception of their decision process and of their reasons for leaving [47]. In addition, the experiences made in the new job might have changed the retrospective perception on their former MA profession. However, this time lag might also have been beneficial, as some former MA reported that they needed some time to be able to fully reflect on their process of leaving (e.g., MA realizing that they have been kept down by their employer).

We applied a convenient sample approach and a wide recruitment strategy. However, we did not recruit any participants with a low educational background. This might be due to a lower participation rate of people with a lower socio-economic status in health studies [48]. It could also imply that in particular MA with a higher level of education might be more likely to leave the MA profession potentially because they feel a higher need for career prospects and perceive to have more alternatives on the job market [49]. Further, we recruited one participant through personal contacts, as recruitment was initially difficult. However, the interviewer and the respective participant did not know each other personally. According to the analyzing author the participant’s responses were in line with those of the other participants and did not indicate any notable deviations. In addition, in terms of a potential selection bias, we excluded MA in training and did not particularly recruit MA who just finished training. Therefore, this study mainly covered perceptions of former MA with several years of work experience. Generational aspects as well as differences of current life phases (e.g., finishing further training, settling down, family planning, children being out of the house) might influence the constellation and weighting of the reasons for leaving [50]. Hence, future research should further focus on MA in sensitive periods of their life, ideally applying a life course approach [51] (e.g. novices on the job or those in the phases of family planning or parenthood). This would allow to follow MA over a long period of time and particularly gain insights on decision making regarding turnover during sensitive periods. It would additionally allow to explore potential differences in the priorities of reasons for leaving the MA profession as well as adequate retention measures.

## Conclusion

This study elucidates the complex process that underlies the decision to leave the MA profession and explores the reasons as well as potential interventions. Reasons for leaving the MA profession were, for instance, a persistently high workload, limited career prospects and poor interpersonal relationships with supervisors and patients. In light of an already existing shortage of MA it seems necessary to counteract this trend. Former MA suggested several potential measures addressing relevant actors (e.g., supervisors, politics, MA themselves), such as to increase the salary, improve work processes, improve career prospects, increase scope of tasks and increase recognition from supervisors, patients and society.

## Supplementary Information

Supplementary Material 1.

Supplementary Material 2.

Supplementary Material 3.

Supplementary Material 4.

## Data Availability

Data cannot be shared publicly because the transcripts contain highly sensitive information (e.g. conflicts with employers and colleagues, mental health). The ethics committee of the medical faculty of Düsseldorf would like to share the data on request only. The category system of qualitative content analysis is available from the corresponding author on reasonable request.

## Footnotes

1 The collective wage agreement for MA is an agreement on the rights and obligations of employees and employers (e.g., salaries, special payments, working hours and vacation entitlement). It is negotiated between the Working Group for the Regulation of Working Conditions of Medical Assistants (AAA) representing outpatient physicians and the Association of Medical Professions (VmF) representing MA. Only if the employer and the MA are a member of the respective association (i.e., AAA or VmF), is the employer obliged to apply the collective agreement. However, membership to the AAA and VmF are voluntary. According to the 2024 collective agreement, the average hourly wage for MA is €16.63 per hour. In comparison, the minimum wage is €12.41 in Germany in 2024.
