# Supplementary material for: Leaving the profession as a medical assistant: a qualitative study exploring the process, reasons and potential preventive measures
Source: BMC Health Serv Res. 2024 Sep 24;24:1111. doi: 10.1186/s12913-024-11607-7 (PMC11423516; doi:10.1186/s12913-024-11607-7)
Supplement: Supplementary file 1 — Supplementary Material 1. [file 12913_2024_11607_MOESM1_ESM.docx]

**Appendix 1**

Table Appendix 1. COREQ checklist.

| No | Item | Guide question/description |  |
| --- | --- | --- | --- |
| Domain 1: Research team and reflexivity | | |  |
| Personal characteristics | |  |  |
| 1 | Interviewer/facilitator | Viola Mambrey |  |
| 2 | Credentials | Viola Mambrey (M.Sc.), Annegret Dreher (Dr.), Adrian Loerbroks (Prof. Dr.) |  |
| 3 | Occupation | VM, AD: research associate; AL: university professor and working group leader |  |
| 4 | Gender | VM, AD: female; AL: male |  |
| 5 | Experience and training | VM: educational background in public health, practical experience in occupational health research  AD: educational background in epidemiology, practical experience in occupational health research  AL: educational background in epidemiology and public health, extensive practical experience in occupational health research, qualitative and quantitative research and teaching |  |
| Relationship with participants | |  |  |
| 6 | Relationship established | No |  |
| 7 | Participant knowledge of the interviewer | Participants knew VM as a researcher in the field of medical assistant health from advertisement of the study and prior publications |  |
| 8 | Interviewer characteristics | No other characteristics were reported about the interviewer |  |
| Domain 2: study design | |  |  |
| Theoretical framework | |  |  |
| 9 | Methodological orientation and Theory | Qualitative content analysis building on Kuckartz |  |
| Participant selection | |  |  |
| 10 | Sampling | Convenience sampling |  |
| 11 | Method of approach | Invitation to the study via email and online distribution of the study call via social media |  |
| 12 | Sample size | 20 participants |  |
| 13 | Non-participation | Not applicable |  |
| Setting | |  |  |
| 14 | Setting of data collection | Participants participated in telephone interviews |  |
| 15 | Presence of non-participants | No |  |
| 16 | Description of sample | 18 female, 2 male; between 26 to 64 years old (M=44.5) |  |
| Data collection | |  |  |
| 17 | Interview guide | Provided as supplemental material |  |
| 18 | Repeat interviews | None |  |
| 19 | Audio/visual recording | Audio recording |  |
| 20 | Field notes | None |  |
| 21 | Duration | 23-66 minutes (mean=38.1) |  |
| 22 | Data saturation | Yes |  |
| 23 | Transcripts returned | No |  |
| Domain 3: analysis and findings | |  |  |
| Data analysis | |  |  |
| 24 | Number of data coders | Two (VM, AD) |  |
| 25 | Description of the coding tree | Yes (in the results section) |  |
| 26 | Derivation of themes | Deductive coding: decision-making process, reasons for leaving the MA profession, motivation for returning to MA profession and preventive measures  All other categories were created inductively |  |
| 27 | Software | MAXQDA 2024 |  |
| 28 | Participant checking | No |  |
| Reporting | |  |  |
| 29 | Quotations presented | Yes |  |
| 30 | Data and findings consistent | Yes |  |
| 31 | Clarity of major themes | Yes |  |
| 32 | Clarity of minor themes | Yes |  |
