# Supplementary material for: Leaving the profession as a medical assistant: a qualitative study exploring the process, reasons and potential preventive measures
Source: BMC Health Serv Res. 2024 Sep 24;24:1111. doi: 10.1186/s12913-024-11607-7 (PMC11423516; doi:10.1186/s12913-024-11607-7)
Supplement: Supplementary file 2 — Supplementary Material 2. [file 12913_2024_11607_MOESM2_ESM.docx]

**Interview guide:**

The text in italics is for the interviewer's guidance.

| **Interview questions** | |
| --- | --- |
| **Decision for medical assistant (MA) profession** | Our topic today is leaving the MA profession. But I would like to start from the beginning. Please tell me why you decided to become an MA back then.  *How did you envision this profession?*  *What were your expectations of the MA profession?* |
| **Reasons for leaving the MA profession/ decision making process** | Before we talk about the reasons why you left the MA profession, I would like to take you back to your old workplace: can you describe a typical working day for me?  And when you think of this typical working day now, what circumstances/reasons led you to leave the MA profession?   - *What other reasons led you to leave the MA profession at that time?*   - *Voluntary/involuntary*   - *Cooperation (superiors, colleagues)*   - *Financial*   - *Reorientation/promotion opportunities*   - *Appreciation/recognition*   - *Enjoyment of the profession*   - *Patient behavior*   - *Reluctant goers/ enthusiastic goers*   - *Ideological aspects*   - *What general conditions influenced your decision to leave the MA profession?*   - *What social aspects led you to leave the profession?* - *What kept you in the MA profession for so long?* - *In which phase of your life did the decision process take place? When did you start thinking about it?* - *Inner resignation: When did you feel that you had already resigned internally? What circumstances/reasons contributed to this?*   What was ultimately the most important factor that made you quit?   - *Were there any specific triggers?* |
| **Career choice criteria** | You are now working in another profession or in another training program.  May I ask what professional activity/training you are currently pursuing?  What exactly made you decide to do this?  *What considerations did you take into account when choosing your new job/training/study program?*   - *Criteria: Working atmosphere, range of tasks, salary, working hours (family-friendliness, work-life balance), flat hierarchies, opportunities for promotion, appreciation, chance?* - *Why did you choose this particular course of study/training?* - *What would you like to do with it later?*   What was the most important criterion for you when choosing your job/training/study program?) |
| **Situation in the new profession** | On a scale of 1-10, how satisfied are you with your current job situation compared to your situation as an MA? One means not at all satisfied and ten means highly satisfied.   - *Why did you choose [number]? /Explain the reasons why you chose [number]?* - *Why did you not choose the [number]? What would have to be fulfilled for you to choose [number]?* - *Which aspects are better now?* - *Which aspects are now worse?* - *Which expectations were fulfilled?* - *Which expectations were not fulfilled?* - *What aspects of your old and new job would make the perfect job for you?* |
| **Motivation to return** | What could motivate you to return to the MA profession?   - *Circumstances, conditions* - *Incentives (public transport ticket, fuel voucher)* - *Payment: how much more would that be roughly?*   If a return is ruled out: What would have motivated you to continue working as an MA at that time? |
| **Prevention approaches/aids for political direction/control** | Generally speaking: In your opinion, what factors would have to change to prevent MAs from migrating to other professions? |

| **Questionnaire** | |
| --- | --- |
| **Sex?** | o Male o Female o Divers |
| **What year were you born?** | _____ |
| **Are you in a relationship?** | O Yes  O No |
| **Your highest school-leaving qualification** | o Finished school without leaving certificate  o Secondary school qualification (‘Haupt-/Volksschulabschluss’)  o Secondary school level I certificate (‘Mittlere Reife’)  o General qualification for university entrance (‘Abitur’) or entrance qualification limited to universities of applied sciences (‘Fachhochschulreife’)  o Other school-leaving qualification (e.g. obtained abroad) |
| **How did you hear about this study?** | O MA cohort study  O Professional organization of medical assistants in Germany  O Personal contacts |
| **How many years have you worked as an MA in total?** | Years |
| **How many months/years ago did you (last) leave your job?** | Month  Years |
| **Where did you last work as an MA?** | O General practitioner  O Specialist  O Other employer, namely: ______________________________ |
| **The following question relates to training as an MA:**  **I would recommend (young) people to train as an MA.** | O Do not agree at all  O Rather disagree  O Agree somewhat  O Strongly agree |
| **If you had to make the decision again, would you choose the profession of MA again today?** | O Definitely  O Probably yes  O Probably not  O Certainly not  O Don't know |
| **Are you currently working in the healthcare sector?** | O Yes  O No |
| **How likely do you think it is that you will return to the MA profession?** | O Very likely  O Likely  O Unlikely  O Very unlikely |
| **Are you more satisfied with your current job than with your job as an MA?** | O Yes  O No  O Neither |
