# Supplementary material for: Leaving the profession as a medical assistant: a qualitative study exploring the process, reasons and potential preventive measures
Source: BMC Health Serv Res. 2024 Sep 24;24:1111. doi: 10.1186/s12913-024-11607-7 (PMC11423516; doi:10.1186/s12913-024-11607-7)
Supplement: Supplementary file 3 — Supplementary Material 3. [file 12913_2024_11607_MOESM3_ESM.docx]

Table A3. Verbatim quotes of former MA.

| Topic |  | Verbatim quote | Participant |
| --- | --- | --- | --- |
|  |  |  |  |
| **The decision-making process** | Q1 | “I had to earn money. So, there was a total interdependency, and one was afraid, or one wasn’t confident enough to say: ‘I’ll find something new in another medical practice.’ It is especially your bosses who convey this feeling of dependency to you: ‘You’ll never find a new job if you quit now.’ This is really exhausting. And as a young woman, you actually believe that and then you’re too afraid to quit.” | ID 7123  Female  Age 59  Years of experience 41 |
| **Reasons to exit from the MA profession** |  |  |  |
| Constant high workload | Q2 | “Every time I was asked why I had quit, I told them that I basically couldn’t handle the vast number of patients, especially due to some of their expectations. I was just getting dull.” | ID 3491  Female  Age 29  Years of experience 5 |
|  | Q3 | “It was also really hard on the phone sometimes. It’s definitely emotionally challenging to say ‘no’ on the phone several times a day, or to countlessly discuss about waiting times.” | ID 3491  Female  Age 29  Years of experience 5 |
|  | Q4 | “I would say it slowly started when you had to do more paperwork. And eventually, it was not about the patients anymore, you just had to fill in more and more documents.” | ID 6912  Female  Age 43  Years of experience 16 |
|  | Q5 | “Why do I have to write a doctor’s letter on my computer, print it out and then put it on a fax machine to send it? That’s nonsense. I could also send an e-mail, or I could transmit it digitally. I don’t have to use ancient methods, to be honest.” | ID 5204  Male  Age 30  Years of experience 5 |
|  | Q6 | “It’s getting more and more. It’s not decreasing and it’s not stagnating either. On the contrary, requirements have increased more and more, but the salary certainly hasn’t. The way we treat each other in the team has also become less pleasant, because we all reach our limit at some point.” | ID 6734  Male  Age 37  Years of experience 10 |
|  | Q7 | “This is also a huge problem in practices. Everything tends to be all-inclusive. That means they expect you to keep overtime hours instead of reducing them, or to not even write them down in the first place.” | ID 3647  Female  Age 59  Years of experience 34 |
|  | Q8 | “Well, many colleagues didn’t / or couldn’t come to work anymore, because they were at their limit, which is completely understandable. […] It was just a physical but also an emotional burden for them.” | ID 2897  Female  Age 33  Years of experience 9 |
|  | Q9 | “We don’t have enough assistants, we’re simply understaffed. We don’t have enough people, and the doctors don’t have enough time for their patients either. We just don’t have enough time for everything. It’s merely stressful, and everyone is annoyed and that’s just an unpleasant work climate.” | ID 5510  Female  Age 37  Years of experience 20 |
|  | Q10 | “And then, the process wasn’t changed at all. Everything had to continue. That means we often had days when we / We were 13 employees in total, I think, and we often had to work with 8. You know? And we were not supposed to cancel anything. And that was just too much, and it happened very often.” | ID 3515  Female  Age 64  Years of experience 42 |
|  | Q11 | “Well yes, if nobody joined you, you didn’t say no either for the sake of your colleagues, you know? So, each time, you swallow the pill. And you might be able to do that for some time, but eventually, you just grow tired of it.” | ID 3515  Female  Age 64  Years of experience 42 |
| Perceived barriers to further training and poor career prospects | Q12 | “So, there’s nothing you can decide for yourself in the end. And that was / (...) Sure, a lot of people would say: "Well, then you don't have the responsibility." I would have liked to have more responsibility. I would have liked to learn more and I would have liked to take on more responsibility. And that was just not possible..” | ID 9162  Female  Age 46  Years of experience 22 |
|  | Q13 | “I don’t want to spend another 20 years printing out prescriptions, talking to people on the phone and making appointments.” | ID 9162  Female  Age 46  Years of experience 22 |
|  | Q14 | “It was quite clear for me that you can’t get any further with the training in a family practice or in a smaller practice in general […]. Which is why I simply changed the profession or, well, the direction.” | ID 8476  Female  Age 26  Years of experience 6 |
|  | Q15 | “The stagnation. In other words, I didn’t have the chance to have more responsibility, or earn more money anywhere.” | ID 9162  Female  Age 46  Years of experience 22 |
| Interpersonal factors |  |  |  |
| Supervisor | Q16 | “And frankly speaking, I work as an unskilled waiter about 5 kilometers away. There, I can cancel at short notice if something else comes up. I have much less responsibility. I mean, it’s not only about responsibility, I just don’t want to do further training in a profession for three years and then have to work for minimum wage.” | ID 8476  Female  Age 26  Years of experience 6 |
|  | Q17 | “Back then, I couldn’t imagine that the money I was getting was very little. So, I didn’t realize that you can’t actually live off the salary of a medical assistant.” | ID 8982  Female  Age 42  Years of experience 23 |
|  | Q18 | “And when you’re waiting tables at the weekend in addition to your regular 40-hour job / which often takes up more time, just to be able to afford some luxuries, then you don’t even have enough time to spend any money at all, and that’s really sad at some point. […] I’m not even talking about incredible luxury goods, just some small things that everyone would like to treat themselves to, somehow.” | ID 6734  Male  Age 37  Years of experience 10 |
|  | Q19 | “I wanted to be able to walk there, because I couldn’t afford a car as a medical assistant. I worked full-time. I could afford the apartment, but having a car is a luxury. You just have to be very clear about that.” | ID 5975  Female  Age 29  Years of experience 6 |
|  | Q20 | “The collective wage agreement is great. But in my experience, practices can’t pay you that much. […] So, I was offered not more than group two, in accordance with my years of professional experience. Whereas I’d actually be in group four or five according to my trainings. And you can’t find any practice that hires you under these conditions.” | ID 6224  Female  Age 42  Years of experience 22 |
|  | Q21 | “For me, it was mostly about the lack of recognition of what you were doing there whatsoever. […] It’s hectic, it’s chaotic and so on, but that has never really been an issue. For me, it has always been the lack of recognition, at least at the end.” | ID 8463  Female  Age 49  Years of experience 15 |
|  | Q22 | “I mean, sure, the physician is the one who is responsible. And he also has to take care of the issue of how the practice is running / He is responsible for the business. And he also has to pay us. But it would be nice to communicate with each other and talk about how things work best here, you know? So that you’d also ask the person sitting at the front desk, right? What could be changed, maybe also with regard to the whole team, you know?” | ID 8463  Female  Age 49  Years of experience 15 |
|  | Q23 | “And then, your boss comes out, and he’s standing behind you, shrugging his shoulders and he tells you: ‘Well, alright, I will do it one more time [note: to write a prescription].’ And you’re sitting there like a fool. […] And the patient gives me the evil eye there. Well, they get the impression that I made something up and that I didn’t want to write the prescription for them. For me, that’s also / there is no support, there is no / Well, that was in fact missing, you know?” | ID 6912  Female  Age 43  Years of experience 16 |
|  | Q24 | “Well, in the practice […] our cleaner quit. And then my colleagues started cleaning the practice. And I was like: ‘Are you crazy?’ I would never do that. Not because I consider myself too good for cleaning. I’ve earned a lot of additional money with cleaning. But they hired me as a medical assistant and not as a cleaner, so there’s a limit there.” | ID 3647  Female  Age 59  Years of experience 34 |
|  | Q25 | “The bosses in the practices don’t want you to talk with patients anymore or to ask questions. They don’t want that anymore. The last thing my bosses said was: ‘Don’t spend so long on the phone. Why would you even ask the patient how he is doing?’ Because it might be important, both for the patient and perhaps also for the appointment? Maybe also because the patient is 80 years old and has been a patient here for many years, and maybe it’s also important for him to briefly talk about his current issues. And that might already help them a lot just because you listened for two minutes. It’s just two minutes. ‘Well, but you don’t get paid for that and we don’t earn any money with that, so would you hang up already?’” | ID 7123  Female  Age 59  Years of experience 41 |
|  | Q26 | “We had to do more and more tasks but nobody cared about the staff.” | ID 9955  Female  Age 51  Years of experience 31 |
|  | Q27 | “I strongly noticed that it’s a business enterprise. But what about the people? And I didn’t like that, I didn’t like that at all, so I could no longer do justice to the patients with my personal attitude eventually.” | ID 2897  Female  Age 33  Years of experience 9 |
| Colleagues | Q28 | “Yes, it’s a typical female profession, it’s a cat fight. Effectively, I did a lot of further training, because I was really interested, I was eager to always learn new things. I wanted to implement a lot as well. And because of that, I rubbed up the wrong way with my colleagues.” | ID 8982  Female  Age 42  Years of experience 23 |
| Patients | Q29 | “Well, effectively, there has been more and more pressure, especially from the patients. The atmosphere among patients has rapidly changed in the last years. They’ve become more and more demanding, also more aggressive. In fact, you weren’t able to please anyone anymore.” | ID 8982  Female  Age 42  Years of experience 23 |
|  | Q30 | “Expectations have become much higher in the last years. That means, they want something, and they want it right now. And I’m not talking about people who lost their arm, you know? […] Rather, these are people who need a prescription, […] They forget about it, but we have to do it right away. And then, you always have to ask yourself: ‘Alright, should I argue with that person or should I just do it?’ And then you try to make your position clear and tell them: ‘Please let us know at least one day in advance.’ Well, somehow you always have to discuss it. ‘Well, can’t you just quickly do it on the side?’” | ID 5975  Female  Age 29  Years of experience 6 |
|  | Q31 | “Well, as a medical assistant on the phone, you’re often / ‘No, I’d rather want to show it to the doctor.’ And that’s where the appreciation goes down the drain, because I’m there to sort things on behalf of the doctor. And then you’re not really taken seriously either.’” | ID 5975  Female  Age 29  Years of experience 6 |
| External factors | Q32 | “Regular practices did not get this bonus [note: single payments during the COVID-19 pandemic]. A friend of mine who is an educator and who was at home due to short-time work received 1.500 euros. My friend on the construction site, who actually had no changes in his work routine at all, also got 1.500 euros corona bonus. And I have to hustle so much every day, I suddenly had to become a corona expert, and I didn’t get a bonus. That’s unbelievable.” | ID 5975  Female  Age 29  Years of experience 6 |
|  | Q33 | “Well, people who were at home got it. (laughs) And I’ve been there for ten hours instead of eight, and people giving me a hard time for ten hours and I simply didn’t get it.” | ID 5975  Female  Age 29  Years of experience 6 |
