# Supplementary material for: Leaving the profession as a medical assistant: a qualitative study exploring the process, reasons and potential preventive measures
Source: BMC Health Serv Res. 2024 Sep 24;24:1111. doi: 10.1186/s12913-024-11607-7 (PMC11423516; doi:10.1186/s12913-024-11607-7)
Supplement: Supplementary file 4 — Supplementary Material 4. [file 12913_2024_11607_MOESM4_ESM.docx]

| Constantly high workload | Perceived barriers to further training and poor career prospects | Interpersonal factors | External factors |
| --- | --- | --- | --- |
| - Causes - High number of patients - Limited time for task fulfillment & adequate patient care - Increasing administrative tasks - Outdated processes concerning bureaucracy - Consequences - Long waiting times for patients - Constant lack of time to complete work - Work stress, poor well-being - Long working hours - Cause and consequence - Staff shortage | - Lack of career prospects - Limited opportunities to apply further trainings - Limited support for further training from supervisors | - Supervisor - Social behavior - Low recognition - low salary, lack of say within practice - Lack of support - Dependency relationship to supervisor - Economic thinking      - Colleagues - Poor team dynamics - Patients - High demanding behavior - Lack of recognition towards MA | - Politics - Legal frameworks (e.g., delegation of task to MA, budgeting of health services) - Society - Low recognition |

Table A.4. Overview of the reasons for MA profession turnover.
